# Periodized resistance training for persistent non-specific low back pain: a mixed methods feasibility study

**DOI:** 10.1186/s13102-020-00181-0

**Published:** 2020-05-08

**Authors:** Svein O. Tjøsvoll, Paul J. Mork, Vegard M. Iversen, Marit B. Rise, Marius S. Fimland

**Affiliations:** 1 Department of Neuromedicine and Movement Science, Faculty of Medicine and Health Sciences, Norwegian University of Science and Technology, Trondheim, Norway; 2 Department of Public Health and Nursing, Faculty of Medicine and Health Sciences, Norwegian University of Science and Technology, Trondheim, Norway; 3 Department of Mental Health, Faculty of Medicine and Health Sciences, Norwegian University of Science and Technology, Trondheim, Norway; 4 Unicare Helsefort Rehabilitation Centre, Rissa, Norway

**Keywords:** Heavy resistance training, Weekly undulating periodization, Persistent non-specific low back pain, Mixed methods, Feasibility study, Numeric pain rating scale, Pain-related disability, Pain self-efficacy, Muscle strength

## Abstract

**Background:**

We investigated the feasibility of a 16-week supervised heavy resistance training program with weekly undulating periodization for individuals with persistent non-specific low-back pain (LBP).

**Methods:**

Twenty-five adults with persistent non-specific LBP participated in this mixed methods feasibility study. Participants trained a whole-body program consisting of squat, bench press, deadlift and pendlay row two times per week for 16 weeks. We assessed pain intensity, pain-related disability, pain self-efficacy and one-repetition maximum strength at baseline, 8 weeks and 16 weeks. Three focus group interviews were conducted at the end of the program. Linear mixed models were used to assess changes in outcomes, and the qualitative data was assessed using systematic text condensation.

**Results:**

We observed clinically meaningful reductions in pain intensity after 8 and 16 weeks of training. The mean difference on the numeric pain rating scale (0–10) in the last 2 weeks from baseline to 8 weeks was 2.6 (95% CI: 1.8–3.6) and from baseline to 16 weeks 3.4 (95% CI: 2.5–4.4). In addition, there were improvements in pain-related disability (3.9, 95% CI: 2.3–5.5), pain self-efficacy (7.7, 95% CI: 5.4–10.1) and muscle strength. In the focus group interviews, participants talked about challenges regarding technique, the importance of supervision and the advantages of periodizing the training. Perceived benefits were improved pain, daily functioning, energy level and sleep, and changes in views on physical activity.

**Conclusion:**

Periodized resistance training with weekly undulating periodization is a feasible training method for this group of individuals with persistent non-specific LBP. A randomized clinical trial should assess the efficacy of such an intervention.

**Trial registration:**

**clinicaltrials.gov****/** Identifier – NCT04284982, Registered on February 24th 2020.

## Background

Low back pain (LBP) is a leading cause for years lived with disability [1–3]. Most patients (> 90%) in primary care have non-specific LBP, meaning that the symptoms cannot reliably be attributed to a specific disease or pathology [4]. Current guidelines recommend non-pharmacological interventions including exercise therapy alongside general activity, cognitive behavioral therapy and education for the management of persistent LBP [2, 5]. Exercise in general can improve pain and function in individuals with persistent LBP [6, 7], and it has been suggested that resistance training in combination with flexibility training can be especially beneficial compared to aerobic exercise, passive treatments and coordination training [8]. Furthermore, a systematic review from 2012 concluded that heavy resistance training with intensities above 70% of one-repetition maximum (1RM) may be more effective than training with lower intensities in reducing pain and improving function in individuals with persistent LBP [7]. Importantly, the review also indicated that heavy resistance training is well tolerated also by individuals with LBP and does not increase risk of injury if the training is increased gradually and carried out in a periodized manner [7].

Periodization of resistance training by systematic structuring of the frequency, volume, intensity and rest, is recommended for healthy individuals to optimize strength gain and reduce the risk of overtraining and injury [7, 9]. Various periodization models exist: undulating periodization comprises a frequent variation in stimuli between low, moderate and high intensity typically on a weekly basis, whereas traditional linear periodization typically contains low load and high volume in the initial phase of training with a gradual shift towards high load and low volume as the training progresses [9].

Little is known about the impact of periodized resistance training in individuals with persistent LBP, but a few small studies have indicated that periodized resistance training might be effective in reducing pain and/or improving function [10–14]. The composition of free weight exercises commonly used by powerlifters (squat, bench press, deadlift and pendlay row) has to our knowledge not been tried in the management of LBP. The current literature indicates that interventions using a whole-body approach is more effective than specific training of trunk muscles [15]. Powerlifting exercises incorporate functional movement patterns with free-weights which could be beneficial for individuals with LBP. Thus, we investigated the feasibility of a supervised 16-week whole-body resistance training program with weekly undulating periodization for individuals with persistent non-specific LBP. Feasibility was assessed through quantitative changes in pain and functioning, and through their qualitative experiences with the programme.

## Methods

This was a mixed methods feasibility study combining quantitative and qualitative methods [16]. The study was approved by the Regional Committees for Medical and Health Research Ethics in Central Norway (REK midt 2017/905), and registered on clinicaltrials.gov/ with the registration number [NCT04284982]. The study was carried out according to the latest revision of the declaration of Helsinki.

All potential participants underwent a clinical examination by a licensed physical therapist, 3 weeks before the intervention started. All participants received written and oral information and signed a consent form before taking part in the study. The participants were informed verbally and in writing about their right to withdraw from the study at any time without stating any reasons to do so.

### Inclusion and exclusion criteria

The inclusion criteria for participation were 1) age between 18 and 65 years, 2) persistent non-specific LBP with a duration > 3 months, 3) average LBP intensity last 2 weeks ≥4 on numerical pain rating scale (NPRS) (0–10 scale), and 4) no experience with heavy resistance training. Exclusion criteria were 1) previous surgery of the low back, 2) radiculopathy, 3) structural spinal changes and/or specific spinal conditions that limit function (spinal stenosis, ankylosing spondylitis, spondylolisthesis/spondylolysis, protrusion, structural scoliosis), 4) autoimmune and systemic inflammatory diseases, 5) cardiovascular disease, 6) neurological diseases and 7) severe osteoporosis.

### Recruitment and clinical examination

Participants were recruited through public internet-based announcements and among students and staff at the Norwegian University of Science and Technology (NTNU). A total of 173 individuals showed interest to participate in the study and the first 37 volunteers that reported that they were eligible for participation were called in for a clinical examination. The clinical examination consisted of assessment of function and tests to rule out severe pathology and to exclude potential participants not satisfying the inclusion/exclusion criteria. After the clinical examination, 25 individuals were found eligible and were enrolled in the study.

### Training program

The 25 participants were divided into 6 training groups of 3–5 participants. Each group trained two times per week with each session lasting about 1.5 h. A physical therapist with experience from powerlifting was present at each session to supervise the participants.

The intervention started with a 4-week adaptation phase to teach the participants correct technique and to reduce potential fear of lifting weights. Exercises were performed with a broomstick the first week. Barbells with very light weights were introduced the second week, and the load was slightly increased in the second training session in the second week. During the last training session in week 3, a 10RM test was carried out to get an impression of 1RM in each exercise. Participants were instructed not to train with a greater range of motion than they could achieve with proper technique and the training load was gradually increased until the participants were unable to perform more than 10 repetitions.

After the adaptation phase, participants proceeded to a resistance training period with heavier weights, using a weekly undulating periodization model with a stepwise progressive overload, divided in 3 cycles – i.e. strength endurance-, strength- and deload cycle (Table 1). During the strength endurance- and strength cycle participants were not supposed to be able to repeat the load from the first set to the following sets so that the decided percentage of 1RM was maintained. For each set the load was reduced by approximately 5%, but without reducing the number of repetitions [17]. Four exercises were performed: Squat, bench press, deadlift and pendlay row with a pronated grip.

**Table 1:** Overview of testing, questionnaires, and strength training throughout the 16 weeks’ intervention period

Week	Tests	Questionnaire	Training emphasis	Set and reps	Pause between sets (min)	% of 1RM
1	Baseline test	NPRS, ODI, PSEQ, FABQ, SNQ	Adaptation	3 x 10	1 – 2	50
2			Adaptation	3 x 10	1 – 2	50
3			Adaptation	3 x 10	1 – 2	50
4	Pretest 1RM		Adaptation	3 x 10	1 – 2	50
5			Strength endurance	3 x 12	1 – 2	70
6			Strength	3 x 8	2	80
7			Strength	3 x 4	3	90
8	Midtest 1RM	NPRS, ODI, PSEQ	Deload	3 x 10	1 – 2	50
9			Strength endurance	3 x 12	1 – 2	70
10			Strength	3 x 8	2	80
11			Strength	3 x 4	3	90
12			Deload	3 x 10	1 – 2	50
13			Strength endurance	3 x 12	1 – 2	70
14			Strength	3 x 8	2	80
15		Qualitative Focus group interviews	Strength	3 x 4	3	90
16	Posttest 1RM	NRPS, ODI, PSEQ	Deload	3 x 10	1 – 2	50

Abbreviations: *NPRS* Numerical Pain Rating Scale, *ODI* Oswestry Low Back Disability Questionnaire, *PSEQ* Pain Self- Efficacy Questionnaire, *FABQ* Fear- Avoidance Beliefs Questionnaire, *1RM* 1 Repetition maximum, *SNQ* Standardized Nordic Questionnaire.

A general and a task-specific warm-up was performed in the beginning of each training session. The general warm-up consisted of low to moderate intensity stair-walking for 5 min. The task-specific warmup consisted of standing unilateral hip flexion, unilateral dynamic stretching and squat using body weight. Additionally, all exercises were performed with light weights, and the weight was then gradually increased until the training load was reached.

### Description of the exercises

Squat: a high bar squat was used with the barbell positioned on the lower part of the trapezius muscle, the pars decendens, just below the 7th cervical vertebrae (vertebrae prominence). The aim was to achieve a depth equal to 110° flexion in the knee joint with the back in a 60–70° angle relative to the floor. The spine was held in a neutral position during the whole range of motion to limit contra nutation of the pelvis, ensuring that an adequate lumbosacral alignment was maintained. The participants received instructions that the line of action had to pass through the midfoot through the whole range of motion to maintain the most optimal barbell path.

Bench press: the starting position was supine on the bench with the nose of the participant directly below the barbell. The barbell had to be just above the shoulder joint in the top position. The participants were instructed to keep the central part (margo medialis) of the scapula as close to the spine as possible while being supine on the bench and throughout the whole set. Grip width was within a range were the participants could keep the forearm almost perpendicular to the barbell in the bottom position. In the bottom position the barbell had to touch just below the lower part of the pectoralis major. The barbell path was a parabola between the bottom and top position.

Deadlift: the starting position was with the barbell placed over the midfoot and the heels of the participants approximately 30 cm apart. The participants were instructed to bend down and grab around the irregular surface of the barbell, parallel to their legs and then move the anterior part of their legs towards the barbell. The barbell had to be in line with the scapula such that the glenohumeral joint was a bit in front of the barbell. The knee joint was in an approximately 100° angle and the spine had to be in a neutral position in an approximately 30° angle compared to the surface. The participants were instructed to press the sternum up to fixate the lumbar part of the spine and to move the inferior part of the scapula (angulus inferior) towards the spine during the whole range of motion. The participants were instructed to use their hips when lifting and to let the spine follow the movement of the hips. The path of the barbell had to be in a straight line and travel along the front side of the legs and thighs throughout the whole range of motion. The participants were instructed to contract their glutes in the top position and avoid hyperextension of the back.

Pendlay row: the starting position was to keep the spine in a neutral position and as close to parallel to the floor as possible. The barbell was resting on the floor between each repetition. A pronated grip with shoulder width was used. The barbell was pulled towards the stomach between the lower part of the chest and above the umbilical. The forearms were kept close to perpendicular to the barbell in the top position. The central part of the scapula was moved towards the spine when the barbell made contact with the stomach. The barbell was then lowered towards the floor for a new repetition.

### Measures for adverse events

In the event of an injury or if any of the participants experienced increased pain during an exercise, the following measures were taken as appropriate: 1) The event was registered and a clinical examination was conducted by the physical therapist that instructed the participants; and if necessary 1) the load was reduced for the respective exercise, 2) the velocity in the movement was reduced, 3) reducing the range of motion and 4) the respective exercise or exercises were stopped for at least a week [18].

### Data collection

Participants completed questionnaires before starting the adaptation phase (baseline), 8 and 16 weeks afterwards. A sample of the participants took part in focus group interviews 15 weeks after starting the training program. Maximal strength for each of the exercises was assessed by 1RM tests in week 4, 8 and 16.

### Questionnaires

Standardized and validated questionnaires were used in the assessment of pain and function. Numerical Pain Rating Scale (NPRS; current pain, pain the last 2 weeks and pain the last 4 weeks), scale from 0 to 10 were 0 equals no pain and 10 equals the worst pain imaginable [19]. A minimum detectable change of 2 points were considered a clinically meaningful change [19]. Oswestry Disability Index [ODI] contains 10 different topics regarding ability to stand, ability to walk, sexual function, sleep quality, ability to travel, intensity of pain, lifting, ability to sit, social life and the ability to care for oneself [20, 21]. The Pain Self-Efficacy Questionnaire [PSEQ] contains 10 items covering aspects such as how well one is coping with pain without medication, household chores, work and social interaction [22, 23]. The Fear Avoidance Beliefs Questionnaire [FABQ] contains 16 items and each item contains a score range from 0 to 6. Higher the scores indicate more fear and avoidance beliefs [24, 25]. The Standardized Nordic Questionnaire [SNQ] contains a map of the body illustrating 9 areas potentially causing pain. In addition, it consists of 4 items regarding whether the individual has had pain > 3 months, whether pain has been present in left and right side of the body, if the pain has compromised the ability to carry out activities of daily living and if the individual has had any previous surgery in their lower back [26].

### Qualitative focus group interviews

After 15 weeks, semi-structured focus group interviews were conducted with three of the six training groups (10 participants). The three groups were chosen randomly by drawing lots. Interviews were based on an interview guide to investigate the participants´ experiences with the training program: the instruction provided, the intensity level and any perceived benefits and challenges. The interviews were conducted by the first author, were audio recorded and transcribed verbatim.

### Quantitative analysis

Outcomes were analyzed according to the intention-to-treat principle and was carried out in STATA/MP version 15.1 for Mac, 2017 (StataCorp, USA). A linear mixed model was used to assess changes in outcomes. All the outcome variables were used as dependent variables and analyzed separately with an interaction of time set as repeated at baseline, 8 weeks and 16 weeks. Cohen’s d effect sizes for changes from baseline to 8 weeks and 16 weeks were calculated from the estimates in the linear mixed models. 0.2, 0.5 and 0.8 were considered small, medium and large effects. Statistical significance was accepted at *P* ≤ 0.05. In addition, we performed per protocol analysis of all outcomes by excluding participants that completed less than 70% of the total training sessions.

To search for confounders a forward approach was used. Basic analyses without any adjustments were conducted first, and then the analysis was adjusted for covariates (gender and age) to see if any confounding effects occurred. If the regression coefficient changed > 10%, it was considered a confounder [27].

### Qualitative analysis

Analysis of the qualitative data was carried out according to systematic text condensation [28] using Nvivo version 11 for Mac. Systematic text condensation is a stepwise approach consisting of four parts: 1) Identifying themes; Temporary themes (Impact on everyday life and perceived change in pain, Exercises and program, Continue with the resistance training) which were identified when reading through the transcription, 2) coding and identifying meaning units; The temporary themes formed the basis for codes which were used as a basis for meaning units, 3) creating subgroups; Every group of codes were divided into subgroups (Carryover effect to everyday life, Pain, Sleep, Social interaction, Energy, Technique, Adaptation, Periodization, View on physical activity and movement, Supervision, Maintain the improvements in pain and function, Motivation), this to demonstrate the nuances in the material. This was further used as a tool for condensation and 4) summarizing and contextualization of the code- and subgroups to produce text units used in the results [28, 29].

## Results

**Fig. 1:**
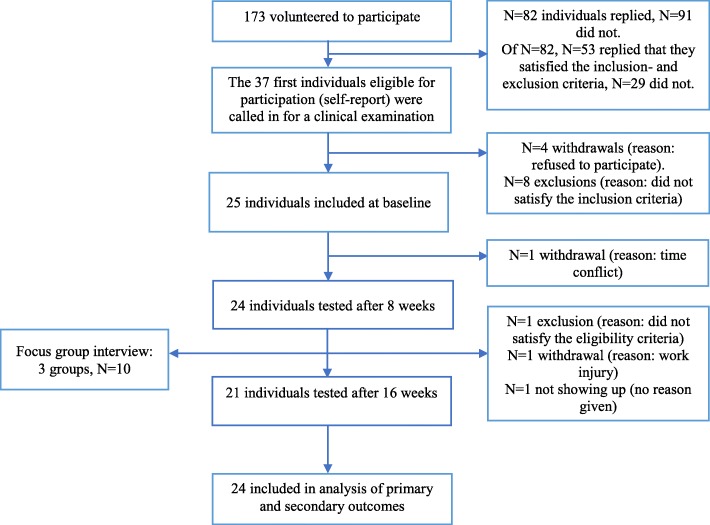
Participant flow throughout the study

No confounding effect was observed from gender or age, and analyses were thus not adjusted. Recruitment started in June 2017 and continued throughout August the same year. Figure 1 illustrates the flow of participants through the study. Out of 25 participants tested at baseline, 24 were included in the intention-to-treat analysis as one individual was excluded due to structural changes in the thoracic spine which had not been detected earlier and thus did not satisfy the eligibility criteria. 23 participants were tested at midtest and 21 at posttest. 20 were included in the per protocol analysis. The drop- out rate from baseline to 8 weeks was 4.2% and from baseline to 16 weeks it was 12.5%.

### Participants characteristics

Baseline characteristics of the participants are presented in Table 2. Mean age was 40 years (range: 22–65 years). Most participants were full- or part time employed, and none were fully or partially sick listed. All the participants had completed either high school or had a university degree. In addition to LBP, most of the participants reported other painful sites. 50% had neck pain, 33% shoulder pain, 29% hip pain, 8% upper back pain and 8% knee pain.

**Table 2:** Baseline characteristics of participants

Participants characteristics	(*n* = 24)
Age, mean (SD)	40 (13)
Women, %	45.8
Married or cohabitant, %	83
Education (High school, college, university), %	100
Employed (full-time or part time), %	87.5
Sick leave (fully/partially), %	0
Pain has prevented you from doing leisure time activities, %	70.8
Neck pain, %	50
Shoulder pain, %	33
Hip pain, %	29
Upper back pain, %	8
Knee pain, %	8
Fear avoidance beliefs questionnaire	
Part A – Activity beliefs (0–24), mean (SD)	8.1 (5)
Part B – Work beliefs (0–42), mean (SD)	9.8 (6.2)

Abbreviations: *SD* Standard deviation*, LBP* Low back pain

### Intention to treat analysis

Changes in outcomes in the intention to treat analysis are presented in Table 3. Mean difference in current LBP on the NPRS from baseline to 8 weeks was 1.3 (95% CI: 0.6–1.9), and from baseline to 16 weeks 1.4 (95% CI: 0.7–2.0). Mean difference in LBP the last 2 weeks on NPRS from baseline to 8 weeks was 2.6 (95% CI: 1.8–3.6) and from baseline to 16 weeks 3.4 (95% CI: 2.5–4.4). Mean difference in NPRS the last 4 weeks from baseline to 8 weeks was 2.5 (95% CI: 1.7–3.4), from baseline to 16 weeks it was 3.2 (95% CI: 2.4–4.1).

**Table 3:** Intention to treat analysis of outcomes, estimated means and 95% confidence intervals at baseline, 8 weeks and 16 weeks

Outcome	Baseline	8 weeks	16 weeks	Effect size (Baseline to 16 weeks)
Outcomes	Mean (95% CI)	Mean (95% CI)	Mean (95% CI)	Cohens d
LBP (NPRS; 0–10)				
Current	3.1 (2.5–3.7)	1.8 (1.2–2.4)*	1.7 (1.1–2.4)*	1.0 (0.5–1.4)
Worst last 2 weeks	6.5 (5.8–7.1)	3.8 (3.1–4.5)**	3.0 (2.3–3.7)**	2.1 (1.6–2.7)
Worst last 4 weeks	6.7 (6.1–7.4)	4.2 (3.5–4.9)**	3.5 (2.8–4.2)**	2.1 (1.5–2.6)
ODI (0–50)	9.2 (7.9–10.5)	7.0 (5.7–8.3)*	5.3 (3.9–6.7)*	1.2 (0.8–1.7)
PSEQ (0–60)	48.8 (46.2–51.4)	54.3 (51.7–57.3)*	56.5 (53.8–59.2)*	1.2 (0.9–1.6)
Squat (kg)	67.8 (58.8–76.8)	84.9 (75.9–93.9)**	103.2 (94.2–112.3)**	1.7 (1.6–1.9)
Bench – press (kg)	56.9 (47.6–66.2)	64.4 (55.1–73.7)**	74 (64.7–83.4)**	0.8 (0.7–0.9)
Deadlift (kg)	77.6 (68.5–86.7)	93.6 (84.4–102.7)**	105.3 (96.7–114.6)**	1.3 (1.1–1.6)
Pendlay row (kg)	49.9 (43.2–56.7)	55.8 (49.1–62.6)**	62.7 (55.9–69.4)**	0.8 (0.7–0.9)

Abbreviations: *NPRS* Numerical Pain Rating Scale*, ODI* Oswestry Low Back Disability Questionnaire*, PSEQ* Pain Self- Efficacy Questionnaire*, LBP* Low Back Pain*, CI* Confidence Interval*.* Change from baseline* *p* ≤ 0.05, ** *p* ≤ 0.01

Mean difference from baseline to 8 weeks in ODI was 2.2 (95% CI: 0.7–3.7), and from baseline to 16 weeks it was 3.9 (95% CI: 2.3–5.5). Mean difference in PSEQ from baseline to 8 weeks was 5.5 (95% CI: 3.3–7.8), and from baseline to 16 weeks 7.7 (95% CI: 5.4–10.1). Moreover, there were improvements in 1RM strength throughout the training period (Table 3).

### Per protocol analysis

20 of the participants completed ≥70% of the training sessions and were included in the per protocol analysis. Only minor differences were observed between the main analysis and the per protocol analysis as presented in Table 4.

**Table 4:** Per protocol analysis (participation > 70) of outcomes, estimated means and 95% confidence intervals from baseline to 8 and 16 weeks

Outcome	Baseline	8 weeks	16 weeks	Effect size (Baseline to 16 weeks)
Outcomes	Mean (95% CI)	Mean (95% CI)	Mean (95% CI)	Cohens d
LBP (NPRS; 0–10)				
Current	2.9 (2.2–3.6)	1.9 (1.2–2.5)*	1.8 (1.1–2.4)*	0.81 (0.3–1.3)
Worst last 2 weeks	6.5 (5.8–7.2)	3.9 (3.1–4.6)**	3.0 (2.3–3.8)**	2.1 (1.6–2.7)
Worst last 4 weeks	6.7 (6.2–7.5)	4.3 (3.5–5.7)**	3.6 (2.8–4.3)**	2.0 (1.5–2.6)
ODI (0–50)	8.4 (7.8–9.7)	7.3 (5.9–8.6)*	5.3 (3.9–6.6)*	1.1 (0.7–1.5)
PSEQ (0–60)	49.1 (51.3–57.1)	54.2 (51.3–57.1)*	56.1 (53.2–59.6)*	1.1 (0.8–1.5)
Squat strength (kg)	65.4 (55.8–75.0)	82.1 (72.5–91.7)**	100.4 (90.7–110.3)**	1.7 (1.6–1.9)
Bench – press (kg)	54.7 (44.4–65.6)	62.4 (51.7–72.3)**	71.7 (61.4–82.1)**	0.8 (0.7–0.9)
Deadlift (kg)	76.4 (66.5–86.8)	92.9 (82.4–103.3)**	104.7 (94.2–115.2)**	1.3 (1.0–1.5)
Pendlay row (kg)	48.3 (41.9–55.7)	53.6 (46.3–61.0)**	60.5 (53.1–67.8)**	0.8 (0.7–0.9)

Abbreviations: *NPRS* Numerical Pain Rating Scale, *ODI* Oswestry Low Back Disability Questionnaire*, PSEQ* Pain Self- Efficacy Questionnaire*, LBP* Low Back Pain*, CI* Confidence Interval*.* Change from baseline* *p* ≤ 0.05, ** *p* ≤ 0.01

### Adverse events

Only one participant reported a slight worsening of LBP and had an increase in LBP from 4 to 5 the last 4 weeks on NPRS.

Two participants experienced minor injuries during the intervention. One participant got a muscle strain in his hamstring at the myotendinous part of the biceps femoris in week 7. No acute symptoms were observed. A clinical examination was performed on the participant to exclude the possibility of a muscle rupture. No signs indicated any severe tear. The participant was not able to perform the deadlift and pendlay row without pain causing compensatory movements for the remaining intervention period. Hence, only squat and bench press were performed thereafter. Because of the injury the participant was only able to perform 1RM bench press at posttest. The other participant got an injury while performing the deadlift in week 9 and a clinical examination indicated a muscle strain in the quadratus lumborum. Because of acute pain and increased tension of the muscle and nearby muscles, the participant was not allowed to continue the training on that day. An examination of the participant’s back was performed during the following training session to ensure no severe injury had occurred. Through modification of the deadlift and the pendlay row the participant was able to perform all exercises. After 3 weeks with an adjusted program, the participant was able to resume the exercises on the desired level of 1RM.

One participant was not able to do the 1RM bench press midtest because of a painful shoulder, and one participant was not able to perform 1RM in the squat at posttest because of LBP.

### Experiences with taking part in the training program

During the group interviews, participants shared their experiences with taking part in the training program, and their perspectives on outcomes and benefits. The main topics covered by the interviews were challenges regarding technique, the importance of supervision, and advantages of periodization.

#### Challenges regarding technique

Several of the participants described that learning the technical aspects of the exercises was difficult. Positioning the body segments was perceived as particularly demanding, as well as understanding the instructions. Most participants described the adaptation phase (i.e., first 4 weeks) as the most challenging part, and that their understanding and execution of exercises progressed throughout the program. Many of the participants said that they initially were skeptical of performing the exercises, especially with heavy load. This skepticism subsided after receiving thorough introduction and being positively surprised by the progression.

*I dare to push myself when lifting […]. It is not dangerous! Before I was skeptical but now it feels good that I dare pushing myself - and nothing bad happens.*

(Chris, 50–55)

Most of the participants said that they found the deadlift exercise challenging. Performing the exercise with proper technique was perceived as difficult – maintaining balance, abdominal bracing and performing the Valsalva maneuver [30], i.e. a special breathing technique used in powerlifting. Many said that they felt a bit frustrated, especially during week 3 and 4, by all the technical details, but that this got better later in the program.

*When I was performing deadlift, I often got feedback that my back and hips did not move in the correct order.* [The instructor] *showed me what I was doing wrong and I saw what he did wrong when demonstrating, but I did not get my body to do the same. I did not understand which muscles I should use to do it correctly... I cannot say that I consciously do anything different now from in the beginning, but now my hips and back move in the correct order. My body has understood it despite not understanding it myself.*

(Meryl, 25–30)

Two of the participants felt that they were struggling when performing squat. They stated that barbell squat was one of the exercises they felt they did not master properly, especially with a heavy load. When performing heavy barbell squats, they experienced LBP but the level of pain did not exceed the pain they had before the program started.

*I feel pain in my back when I am descending and then something happens mentally. I am afraid of descending and that I will not be able to fixate enough. I am not sure what happens but I find that really challenging.*

(Rebecca, 40–45)

Four participants reported that they found it difficult to maintain correct body position in bench press and some found it to be the most difficult exercise. In the initial phase, they found it difficult to lay supine on the bench because of stiffness in the lumbar spine.

*I felt that I was struggling a lot to master the exercises. Especially in bench press to press the shoulder blades together. This is something I still have to work with. I perceived it as mentally challenging to understand what to do.*

(Joseph, 50–55)

Some of the participants described that they initially felt that 4 weeks focusing on technique and using light weights was unnecessary, since they had previous experience with low intensity resistance training. However, as the program progressed they appreciated the value of the adaptation phase as it made them understand the lifting mechanics more and reduced the fear and avoidance towards certain movements. Several participants reported that after the adaptation phase they managed to perform the exercises more or less consistently in every repetition and to a greater extent detect nuances of the movements throughout the range of motion.

#### The importance of supervision

The participants emphasized the importance of supervision, describing this as a crucial element. Several reported that they would have considered to not participate if they had just been given a short introduction and a sheet with exercises.

*I would have tried it, but I know now that I would never have been able to do it correct technically. I would never have lifted as heavy and I would probably have stopped after a short period.*

(Meryl, 25–30)

Many participants mentioned that the small group size was positive and a prerequisite for receiving proper individualized supervision and feedback. Another aspect pointed out by the participants was that they would not have dared to use heavy weights or push themselves if they had to perform the training sessions without supervision and feedback. Knowing qualified personnel was present during training made the participants feel safer and more confident.

*I would never challenge myself as much without knowing or getting feedback along the way that what you are doing is actually correct.*

(Leon, 20–25)

#### The advantages of periodization

All participants described positive experiences with the variation in volume and stimuli in the training program. The variation from week to week was perceived as motivating and preventing the training from becoming monotonous. Some participants said that particularly the higher repetition sets were very demanding, and that heavier weights for fewer repetitions, actually were easier on the body.

*If we were only given series with 12 repetitions I wouldn’t have been able to walk the last couple of weeks. After the sessions with 12 repetitions, I am unbelievably tired. I can feel it when I am going home, my head feels heavy, and the only things you’re able to think is that you have to move the right foot and then the left foot. When we’re performing 8 and 4 repetitions our bodies don’t become nearly as exhausted as when we’re doing 12 repetitions.*

(Chris, 50–55)

Several participants said that they first thought that to perform the same exercises every training session would get boring. However, the variation in load and repetitions constituted a motivating factor. Many participants said that it gave a sense of achievement to experience that it was possible to perform the exercises with heavy load with few repetitions, as well as lower load with high repetitions.

*I think it has been really good, because then you can set new goals as well as noticing that you are challenging yourself. You are moving the boundaries for each cycle. You realize that you are actually able to lift heavier weights without anything bad happening.*

(Lara, 40–45)

#### Perceived outcomes and benefits from the program

The participants described several benefits from taking part in the training program. They talked about different improvements regarding pain, daily functioning, energy level and sleep, as well as changes in their views on physical activity and training.

#### Changes in pain

Whereas some of the participants described minor or no changes, others said that their LBP had changed a great deal. Some described that their LBP had almost disappeared during the training program.

*…I notice that before being part of the program I used to feel tension in my lower back when I was sitting a lot at work. I haven’t noticed this anymore and that probably means that it is gone or that it isn’t present to the same extent as before.*

(Lara, 40–45)

A few of the participants said that although their LBP did not change much during the program, they described being able to stand for a longer time with less pain. In addition, some described being able to handle the pain a lot better, noticing less fear and avoidance of movements. Some participants experienced increased symptoms during the first weeks with periodized strength training, but that this gradually declined and became better than before the program started. Others experienced that their symptoms were undulating during the program.

*It has varied a little during the training period. One period I was almost completely pain free and was shocked how that was possible. But then it became a bit worse again. Still, since the day we started with the training I haven’t experienced pain coming from my lower back going down the backside of my thighs.*

(Meryl, 25–30)

#### Improved daily functioning

Participants described that the improvement in strength made activities in everyday life easier than before. Regarding daily home chores usually inducing LBP, several reported that they managed to perform tasks with reduced or no pain. Tasks such as freshening up morning and night, vacuum cleaning, or cleaning the house, or carrying groceries and heavy objects were described as less challenging.

*We had to buy a new washing machine. It had to be carried into our house and my husband said he had to call a friend for assistance. However, this time I insisted on carrying it together with him and I managed to carry it without any back pain. Surprisingly, it even felt easy.*

(Rebecca, 40–45)

Participants also reported that previous difficulties getting out of bed in the morning due to LBP had improved during the training program. In addition, some said that they now could be more active with their children.

*I’m not sure if it’s the training but I notice that I have become a lot more active with the kids. I want to play with them and I actually have the capacity to do so. Previously, my kids had to walk on my back and give me a massage before I was able to play with them. This is something they don’t have to do anymore.*

(Zelda, 30–35)

Two of the participants reported that in terms of general physical activity, it had not changed their view but that their view on lifting heavy objects had changed drastically. It was something they previously considered unwise, and they not were capable of doing because of fear.

*My view on lifting heavy loads have changed. I used to believe that it was something that was not very smart to do and something I never could do. However, my view on physical activity in general has not changed. Lifting as heavy as we did with low back pain was something I never thought was possible.*

(Meryl, 25–30)

Others expressed that they now dared to try other activities. Leisure time activities such as jogging - and especially downhill jogging – were described as less painful or pain free after going through the training program.

#### Improved energy and sleep

Whereas some of the participants described still feeling tired and with no change in energy level, others felt that their energy level had increased. Several said that the energy surplus made them seek out other activities in their leisure time. Others described that the intervention made them feel fatigued in the first 6 weeks of periodized training. They perceived it as temporary and felt more energized towards the second half of the program. Many were also surprised over how well the body handled the high load and that the body was able to recover between the training sessions.

*I really want to train more and that is a feeling I have not felt in a really long time. Despite being tired, I have started to walk and run because of increased energy and I really enjoy it.*

(Zelda, 30–35)

Half of the participants said they experienced improved quality of sleep throughout the study. Common for the participants that had experienced reduced quality of sleep was that the LBP had disturbed their sleep to such an extent that they had to get up during the night to do light physical activity. For many, this changed significantly during the training program with lower pain intensity and frequency, allowing them to sleep throughout the night.

*I have had a lot of pain in my back during the night or I have woken up in the middle of the night and experienced pain in my back. I have had to get up and move around in order to be able to go to sleep again because of tension in the back. It is still present but a lot less prominent and not nearly as frequent. Now I can sleep through a whole night and I cannot remember the last time I did that before.*

(Lara, 40–45)

#### Continuing resistance training

When asked whether they would continue with the resistance training after the program, all participants replied that they wanted to maintain the training to some degree. Some said that training two times a week would be difficult to maintain due to a busy life, but that they would try to keep up the training whenever it was possible. Most participants said that they had to continue training since they already had invested a lot in completing the program. Some said that they felt committed to maintain the training because of the noticeable improvements in pain and functioning.

*I really want to continue and maintain the improvements in pain and function when I have become so much better in such a short period. I have invested so much time in this that I do not want it to disappear.*

(Jill, 40–45)

Several of the participants reported that it would be challenging to continue the training by themselves. They said that this meant that they would not focus on lifting heavier, but increase the load at a slower pace and have more long-term goals. Some participants also mentioned that in addition to the resistance training they wanted to add some other exercise modalities to maintain their motivation.

## Discussion

In this mixed methods feasibility study, we investigated the feasibility of heavy periodized resistance training with weekly undulating periodization for individuals with persistent non-specific LBP. There was a clinically meaningful reduction in LBP from start to 8 and 16 weeks of follow-up and significant improvements in pain-related disability, pain self-efficacy and muscular strength. In the focus-group interviews, participants highlighted the challenges regarding technique, the importance of supervision, and advantages of periodizing the training. Perceived benefits were improvements in LBP, daily functioning, energy level, sleep, and a changed view on physical activity. Participants also reported challenges regarding continuance of the resistance training without supervision.

Some limitations must be acknowledged. The lack of a randomized control group prevents us from distinguishing between the effects of periodized resistance training and the attention and reassurance provided by the instructor. Second, selection bias is likely as individuals with high motivation and a relatively high level of functioning probably were more likely to respond positively to the advertisement. That all participants had high school, college or university education and none reported to be sick listed suggests this was the case. To reduce selection bias, the first 37 eligible individuals were invited to a clinical examination, rather than just choosing from the applications (e.g. based on motivation). Third, a physical therapist and powerlifter (first author), performed the recruitment, clinical examination, supervision of training, and led the focus group interviews. This may have influenced the results, as the participants might have felt more reassured concerning the safety of training, and thus perform better (experimenter effect). This might also explain the low dropout rate in the study. Moreover, participants could also be hesitant to talk about perceived negative aspects during the interviews, though they were encouraged to be open about this. In addition, only 3 of the 6 training groups were drawn to participate in the focus group interviews. Not including individual interviews could be considered a limitation as they exclude the likelihood of being influenced by other group members, they are more personal, and critical thoughts are more likely to be expressed. Still, an advantage with group interviews is that it facilitates discussion and interaction.

The mechanisms resulting in a clinical meaningful reduction in LBP, and improved pain-related disability and pain self-efficacy are likely multifactorial. The qualitative interviews provide some insight. The participants viewed an extended familiarization phase and close supervision by a competent instructor as very important, reducing the fear of physical activities and heavy lifting in particular. Furthermore, all participants felt that increased strength made their daily life activities easier. This could be due to the four multiple joint exercises used, mimicking activities such as lifting, pulling and pushing, activating all major muscle groups in a functional way. A higher physical capacity in these movements would reduce the effort required to perform the same task, from before to after the program, possibly making them more resilient. In addition, the weekly undulating periodization regimen was described positively. Sets of higher repetitions (i.e. 12) were described as physically and mentally exhausting, while lower repetition-ranges (i.e. 4 and 8) did not have such an impact. Thus, it was well received that the loading was being undulated, as supported by a recent systematic scoping review [31]. This finding could have some clinical relevance as clinicians and patients anecdotally perceives exercises using heavy load as contraindicated for LBP. It is also possible that the number of synergists activated could be of importance. It has been indicated that using barbell squat reaching 80% of 1RM and heavy deadlift elicit greater activation of trunk muscles than isometric instability exercises [32]. Studies that have investigated the fiber type properties of the erector spinae have shown a greater amount of fiber type 1 relative to fiber type 2a and 2x [33], which may indicate that the erector spinae is working more optimally when its performing isometric tasks as the exercises used in the present study requires.

Only a few previous studies have used periodization and heavy resistance training for LBP [10–14]. These studies demonstrated positive indications on outcomes such as pain and function and this is also supported by this study. In addition, two of the studies included high intensity deadlift as the primary exercise. However, there are some differences that makes this study different from the previous studies. In contrast to the previous studies that used linear (traditional) periodization [12–14], we used a weekly undulating periodization model. It has been proposed that undulating periodization of training is more beneficial than traditional periodization as the variation in stimuli with low, moderate and high intensity and recovery is more frequent than in the latter [34–36].

The studies using deadlift suggested that this exercise could be used safely in individuals with LBP and that this exercise could elicit a clinically meaningful change in pain comparable to low load motor exercises [10, 11]. The present study supports these findings as positive indication was seen in pain and function using high load lifting exercises. In contrast to the studies using only deadlift, we incorporated several high load lifting exercises stimulating several movement patterns. The higher intensity in the present study is also dissimilar to previous studies. These differences could have both physiological and psychological benefits. The incorporation of undulating periodization, higher load free weight exercises as well as higher intensity could potentially have greater implications on physical capacity. Positive psychological benefits of these differences could be related to fear and avoidance behavior and self-efficacy, perhaps influencing these even more than previous studies.

Changes in sleep, energy and view on physical activity was not assessed through questionnaires, but still reported to improve by several participants in interviews. Sleep has been shown to be impaired in individuals with LBP [37]. A positive impact on quality of sleep could mediate some of the improvements in pain and function in this study. Conversely, less pain could also allow for more and better sleep. This is in line with a systematic review suggesting that resistance training improves the quality of sleep [38], and it has also been suggested that exercise can reduce the probability of insomnia in individuals with musculoskeletal pain [39]. Moreover, many experienced that as a result of increased energy level they could engage in activities that they normally wouldn’t because of pain and feeling fatigued. The training conducted in the present study might mediate participation in other beneficial activities in their leisure time, potentially improving their level of functioning further.

## Conclusion

In this study, we investigated the feasibility of a 16-week periodized resistance training program, starting with very light loads in the first 4 weeks and then utilizing heavy loads in a weekly undulating periodization model for 12 weeks. We observed clinically meaningful reductions in LBP, and improvements in pain-related disability, pain self-efficacy and muscular strength. The importance of familiarization, regular and competent supervision, and periodization was highlighted as key features of the program by the participants. Only a few minor negative events occurred. Thus, the resistance training program presented in our study appears to be a feasible training method for men and women with persistent non-specific LBP. These positive indications should be investigated further in a RCT.

## Data Availability

Collected data and research protocol are available from the corresponding author on reasonable request.

## Abbreviations

LBP: Low back pain
1RM: One-repetition maximum
NPRS: Numerical pain rating scale
NTNU: Norwegian University of Science and Technology
ODI: Oswestry Disability Index
PSEQ: Pain Self-Efficacy Questionnaire
FABQ: Fear Avoidance Beliefs Questionnaire
SNQ: Standardized Nordic Questionnaire
SD: Standard deviation
